# Special Issue “Resistance to Therapy in Ovarian Cancers”

**DOI:** 10.3390/ijms27031207

**Published:** 2026-01-25

**Authors:** Eros Azzalini, Serena Bonin

**Affiliations:** DSM—Department of Molecular Sciences, University of Trieste, 34149 Trieste, Italy; eazzalini@units.it

## 1. Editorial

Epithelial ovarian cancers (EOCs) are a heterogenous group of neoplasms at the clinical, pathological, and molecular levels. Approximately 70% of EOCs are high-grade serous ovarian cancers (HGSOCs), while the less common epithelial subtypes are endometrioid, clear cell, low-grade serous, mucinous, and carcinosarcoma [1,2]. These latter subtypes are relatively rare, creating major challenges for conducting adequately powered, histotype-specific clinical trials, which contributes to the slow development of evidence-based personalized strategies [3]. Even where molecular alterations are known [4], effective targeted therapies are limited, and precision agents validated in HGSOCs show minimal or unproven benefit in non-HGSOC tumors [3]. Additionally, intrinsic and acquired resistance to chemotherapy and new targeted agents remains frequent in these subtypes, as emphasized in clinical guidelines [5].

With the exception of endometrioid ovarian and clear cell carcinomas, which often present at an earlier stage, most EOCs are diagnosed at advanced stages (III and IV) due to their non-specific symptoms, with a mean 5-year overall survival rate of approximately 47% [6,7,8]. The standard treatment for advanced-stage ovarian cancer consists of cytoreductive surgery, platinum-based chemotherapy, and targeted maintenance therapies such as bevacizumab and/or PARP inhibitors [1,5,9]. Currently, there are no recommended screening methods for EOCs in average-risk, asymptomatic women, and multiple large randomized controlled trials have demonstrated that annual screening with transvaginal ultrasound, CA-125 testing, or multimodal approaches does not reduce ovarian cancer mortality [1,10].

Platinum-based therapy remains the cornerstone of treatment for EOCs in both frontline and recurrent settings, and maintenance strategies with PARP inhibitors (e.g., olaparib, niraparib) are offered to patients who respond to platinum-based therapy [1,5,11]. However, the clinical utility of platinum-based therapy is limited by severe systemic toxicities—affecting the kidneys, heart, liver, and inner ear—and by the frequent emergence of resistance [12,13]. The efficacy of platinum is particularly pronounced in tumors with BRCA mutations or homologous recombination deficiency (HRD), which are more sensitive to DNA-damaging agents. Nevertheless, most patients eventually develop platinum resistance, which is associated with poor prognosis and limited therapeutic options [14]. Such resistance mechanisms include altered drug transport, enhanced DNA repair, epithelial–mesenchymal transition, immune evasion, and changes in apoptosis and autophagy pathways [12,15,16,17]. To overcome these hurdles, research is focused on developing less toxic cisplatin analogs and exploring combination strategies with other drugs, natural compounds, hyperthermia, or radiotherapy [12].

In this Special Issue, several mechanisms and biomarkers implicated in resistance to platinum-based therapy are presented. Among them, matrix metalloproteinase 3 (MMP3) has been associated with resistance and poor prognosis in high-grade serous ovarian cancer [18]. siRNA-mediated MMP3 knockdown was reported to reduce proliferation and invasion in cisplatin-resistant cells, showing synergistic effects when combined with cisplatin [18]. Mortalin, a chaperone involved in proliferation and migration, has also been reported to contribute to cisplatin resistance. In vivo, cisplatin combined with miR-200c, which downregulates mortalin, markedly improved therapeutic outcomes, suggesting miR-200c as a promising adjunct strategy [19].

The introduction of PARPis as maintenance therapy following first-line chemotherapy has ushered in a new era in the management of advanced high-grade serous and high-grade endometrioid ovarian cancer, delivering unprecedented benefits for patients with BRCA1/2-mutated tumors, as well as those with BRCA1/2 wild-type but HRD-positive disease [20,21]. Nevertheless, platinum resistance can manifest in later lines after PARPi treatment due to restoration of HR function [14]. Intratumor heterogeneity profoundly impacts therapy resistance in EOC by creating genetically and phenotypically diverse subclones that can independently adapt to microenvironmental stresses and survive therapy. EOC cells also exhibit inter- and intramechanical variability from a biophysical perspective, suggesting that biomechanical profiling could identify new vulnerabilities and molecular targets for therapy [22,23].

In the absence of screening programs, EOCs urgently require reliable biomarkers to guide therapy response and the development of new therapeutic strategies, particularly for platinum-resistant HGSOCs and other less frequent EOC subtypes. In this context, radiomics has recently emerged as a promising tool, offering noninvasive, quantitative imaging biomarkers that markedly improve the prediction of treatment response [24,25]. When integrated with clinical or genomic variables, radiomics models outperform traditional clinical assessments, achieving substantially higher accuracy, such as an AUC of 0.78 versus 0.47 for clinical assessment alone [26]. Parallel to advances in biomarker development, novel treatment strategies for platinum-resistant EOCs are needed [27]. This includes the use of mirvetuximab soravtansine targeting folate receptor alpha (FRα)-positive cancers, antibody–drug conjugates for select molecular subtypes, and investigational agents targeting immune, angiogenic, and oncogenic pathways [1,28,29,30]. Although immune checkpoint inhibitors targeting PD-1 or its ligand PD-L1 have achieved considerable success in several malignancies, their activity as monotherapy in epithelial ovarian cancer has been modest, with meaningful responses primarily limited to the rare subset of tumors exhibiting microsatellite instability-high (MSI-H) status [29,31]. However, the potential benefit of integrating checkpoint inhibitors into standard treatment regimens, particularly in combination with PARP inhibitors, with or without bevacizumab, remains uncertain and is currently being evaluated in ongoing clinical trials [32].

In recurrent EOCs, the standard care regimen remains non-platinum chemotherapy with or without bevacizumab, but clinical trial enrollment and biomarker-driven therapy are critical to advancing outcomes in this challenging setting [5].

Recently, onvansertib, a polo-like kinase 1 inhibitor acting as an ATP competitor [33], has shown promise in platinum-resistant settings. In patient-derived xenograft models with acquired or intrinsic cisplatin resistance, combinations of onvansertib with gemcitabine or carboplatin were well tolerated and significantly improved survival compared to monotherapies. Mechanistically, these combinations induce higher DNA damage, supporting their clinical translatability [34]. Collectively, these findings underscore the complex nature of platinum resistance and the need for integrated strategies to restore drug sensitivity and improve patient outcomes.

## 2. Conclusions

Platinum-based chemotherapy remains the backbone of treatment for epithelial ovarian cancers, yet its long-term efficacy is hindered by systemic toxicities and the development of resistance. The complexity of resistance mechanisms—ranging from genetic and epigenetic alterations to biophysical heterogeneity—underscores the urgent need for innovative therapeutic strategies. Advances such as PARP inhibitors, antibody–drug conjugates, and emerging agents like onvansertib offer promising avenues, particularly when guided by biomarker-driven approaches. Moving forward, integrating molecular profiling, functional assays, advanced AI-driven multimodal analytics [25], and novel combination therapies will be essential to overcoming platinum resistance and improving survival outcomes. Collaborative efforts between researchers and clinical practitioners are critical in order to translate these discoveries into personalized, effective treatments for patients with advanced and recurrent ovarian cancer.

## Data Availability

No new data were created or analyzed in this study. Data sharing is not applicable to this article.

## Abbreviations

The following abbreviations are used in this manuscript:

EOC	Epithelial Ovarian Cancer
BRCA 1/2	Breast Cancer Gene 1/2
HGSOC	High-Grade Serous Ovarian Cancer
HRD	Homologous Recombination Deficiency
MMP3	Matrix Metalloproteinase 3
MSI-H	Microsatellite Instability-High
PARPi	Poly(ADP-ribose) Polymerase Inhibitor
PD-1	Programmed Cell Death Protein 1
PD-L1	Programmed Death-Ligand 1
siRNA	Small Interfering RNA
